# Visual acuity and stereopsis across the parafoveal and perifoveal retina in young adults: an eccentricity and meridian analysis

**DOI:** 10.7717/peerj.21251

**Published:** 2026-04-27

**Authors:** Bo Yu, Lu Liu, Ning Yang, Lingxian Xu, Huang Wu

**Affiliations:** 1 Department of Optometry, The Second Hospital of Jilin University, Changchun, China; 2 Department of Ophthalmology, Henan Provincial People’s Hospital, Zhengzhou, China

**Keywords:** Visual acuity, Stereopsis, Eccentricity, Meridian

## Abstract

**Background:**

Visual acuity (VA) and stereoacuity (SVA) are fundamental visual functions that decline with increasing retinal eccentricity. Patients with macular degeneration and other central vision disorders often rely on paracentral vision, yet location-specific reference data for VA and SVA across the parafoveal and perifoveal retina remain limited. This study aimed to quantify the distribution of binocular VA and SVA across eccentricity and meridian in young adults, develop prediction equations with 95% prediction intervals, and examine the relationship between these two visual functions in the paracentral retina.

**Methods:**

Thirty-five healthy young adults (13 males, 22 females; mean age 27.23 ± 2.43 years) were recruited. Binocular VA and SVA were measured at 48 test positions across eight meridians (0°, 45°, 90°, 135°, 180°, 225°, 270°, 315°) and six eccentricities (2.5° to 15° in 2.5° increments) using a polarized 3D display system. A four-alternative forced-choice task was employed with tumbling E optotypes for VA and random-dot stereograms for SVA. Eye tracking ensured fixation stability throughout testing. Generalized estimating equations were used to analyze the effects of eccentricity and meridian. Linear mixed-effects models and Bayesian Tobit models were employed to develop prediction equations. Ten-fold cross-validation assessed model generalizability.

**Results:**

Both VA and SVA significantly declined with increasing eccentricity (*P* < 0.001). VA decreased from a median of 0.40 logMAR at 2.5° to 1.20 logMAR at 15.0°, at a rate of 0.057 logMAR per degree. SVA increased from 2.1 log arcsec at 2.5° to 2.9 log arcsec at 7.5°, declining approximately three times faster than VA (0.154 log arcsec per degree). Both functions showed significant meridional anisotropy (*P* < 0.001), with the horizontal meridian demonstrating 0.058 logMAR better VA and 0.106 log arcsec better SVA compared to the vertical meridian. Despite parallel declines with eccentricity, no significant correlations were observed between VA and SVA at any test position within 7.5° eccentricity (*P* > 0.05).

**Conclusions:**

VA and SVA deteriorate with increasing eccentricity in the paracentral retina, with stereopsis declining approximately three times faster and demonstrating a more pronounced horizontal-over-vertical advantage. The absence of correlation between VA and SVA suggests distinct neural mechanisms underlying these functions. The prediction equations with 95% prediction intervals provide reference benchmarks for healthy young adults, facilitating clinical interpretation of patient measurements and enabling objective assessment of disease-related changes in paracentral visual function.

## Introduction

In human vision, two foundational attributes stand out for their essential roles in shaping our perceptual experiences: visual acuity (VA) and stereoacuity (SVA). VA quantifies the smallest spatial detail that can be resolved (Davson, 1990), whereas SVA is a measure of the visual system’s ability to detect the smallest binocular disparity that produces a sensation of depth (Howard & Rogers, 2012). The retina is organized into eccentricity regions: the central fovea (inside 1°), the parafovea (∼8°), and the perifovea (∼18°), the latter two together forming the paracentral field (Swanson & Fish, 1995). Patients with macular degeneration, strabismus or amblyopia often rely on this paracentral vision for mobility, and peripheral stereopsis can still improve hand placement and posture even when foveal function is lost (Verghese, 2023). Hence, location-specific reference data for both VA and SVA in the paracentral retina are clinically important.

The related research consistently demonstrated a decline in VA as the distance from the fovea increases. Carrasco, Williams & Yeshurun (2002) examined visual performance in an acuity task using Landolt squares presented at varying eccentricities along the horizontal and vertical meridians. Their results showed that a decrease in recognition accuracy with increasing eccentricity, with performance deteriorating more rapidly along the vertical meridian than the horizontal. Rees et al. (2005) reported a decrease in VA among seven subjects with normal vision as peripheral retinal eccentricity increased, noting that, with the exception of a 5° eccentricity, VA along the horizontal meridian consistently outperformed that of the vertical meridian. De Lestrange-Anginieur & Kee (2020) further corroborated these findings, concluding that the minimum angle of resolution in the inferior retinal quadrant is significantly worse compared to that in the horizontal meridian. These perceptual declines reflect the underlying structure of the visual system: receptive field size increases with eccentricity across visual areas (Smith et al., 2001), and the cortical magnification factor decreases from the fovea toward the periphery (Rovamo & Virsu, 1979), leading to lower resolution in peripheral vision.

In concordance with the trends observed in vision, stereopsis in parafoveal or perifoveal regions also demonstrated a decline with increasing eccentricity. Rawlings & Shipley (1969) reported stereoacuity decreased from 20″ to 193″ from central fixation to 6° eccentricity. Mochizuki et al. (2012) extended the measurement to eight meridians in 16 observers and reported mean SVA of 474″ ± 202″ at 10°, 725″ ± 704″ at 20°, and 1,223″ ± 1,101″ at 30°, confirming a nearly logarithmic decline. This decline in stereopsis with eccentricity is further explained by Wardle et al. (2012), who demonstrated that increased internal noise, rather than reduced sampling efficiency, limits peripheral depth discrimination.

Previous studies have demonstrated that VA and SVA are not uniformly distributed across the visual field. Both parameters decrease as eccentricity increases and also change depending on the meridian (Dormegny et al., 2023; Ghahghaei, McKee & Verghese, 2019; Himmelberg, Winawer & Carrasco, 2023). Given these asymmetries, it is crucial to investigate how VA and SVA diminish from the fovea as a function of both eccentricity and meridian. VA is the most frequently considered vision-related parameter affecting stereopsis (Okamoto et al., 2020; Okamoto et al., 2021). A reduction in VA could impair stereopsis, and correlations between these two parameters have been observed (Bosten et al., 2015; Lam et al., 1996; Levy & Glick, 1974; Liu et al., 2024; Sitko et al., 2016). Whether the same relationship holds in the parafovea and perifovea in healthy adults remains unexplored. This gap motivates the present study, which provides a location-specific map of VA and SVA across the paracentral retina. Considering the obvious impact of VA and stereopsis on quality of life (Okamoto et al., 2021; Taipale et al., 2019), comprehending their distribution across the parafoveal and perifoveal regions of the retina holds implications for the rehabilitation of patients with central vision loss and the early identification of individuals with damage in these specific retinal areas.

We tested the hypotheses that VA and SVA in the paracentral retina can be predicted by eccentricity and meridian in young adults. Although the age range is restricted, establishing precise reference maps in this population is essential for distinguishing age- and disease-related changes from normal peripheral variations in clinical populations. This comparison will help explain whether disease-related vision loss follows the same eccentricity-dependent patterns as normal peripheral vision, or whether pathological processes disrupt these fundamental spatial relationships.

While previous studies have described the decline of VA and SVA with eccentricity (Blakemore, 1970; Fendick & Westheimer, 1983; Shipley & Popp, 1972; Siderov & Harwerth, 1995), few have mapped SVA across both eccentricity and meridian to establish reference surfaces for the parafoveal and perifoveal retina in healthy young adults. This study provides spatial maps of VA and SVA and tests whether eccentricity and meridian together predict these functions with mathematical precision, offering a reference baseline for interpreting clinical tests in conditions such as early macular disease.

## Materials and Methods

### Participants

A total of 35 participants (13 males and 22 females, mean age of 27.23 ± 2.43 years, age range of 22–33 years) were prospectively recruited in this study. Inclusion criteria included refractive error not exceeding ±3D sphere and ±1D cylinder, anisometropia not excessing 1.50DS or 1.00DC, no history of strabismus or other eye diseases, a best-corrected VA of 0 logMAR or better, and stereoacuity of no worse than 40″ (Stereo Fly Test, Stereo Optical Company, Illinois, USA). Participants were excluded if they reported systemic or retinal diseases or had undergone previous ocular surgery. Before participation in the study, each subject provided written, informed consent. This study adhered to the Declaration of Helsinki and was approved by the ethics committee of the Second Hospital of Jilin University (No. 2024-145).

### Apparatus

**Figure 1:**
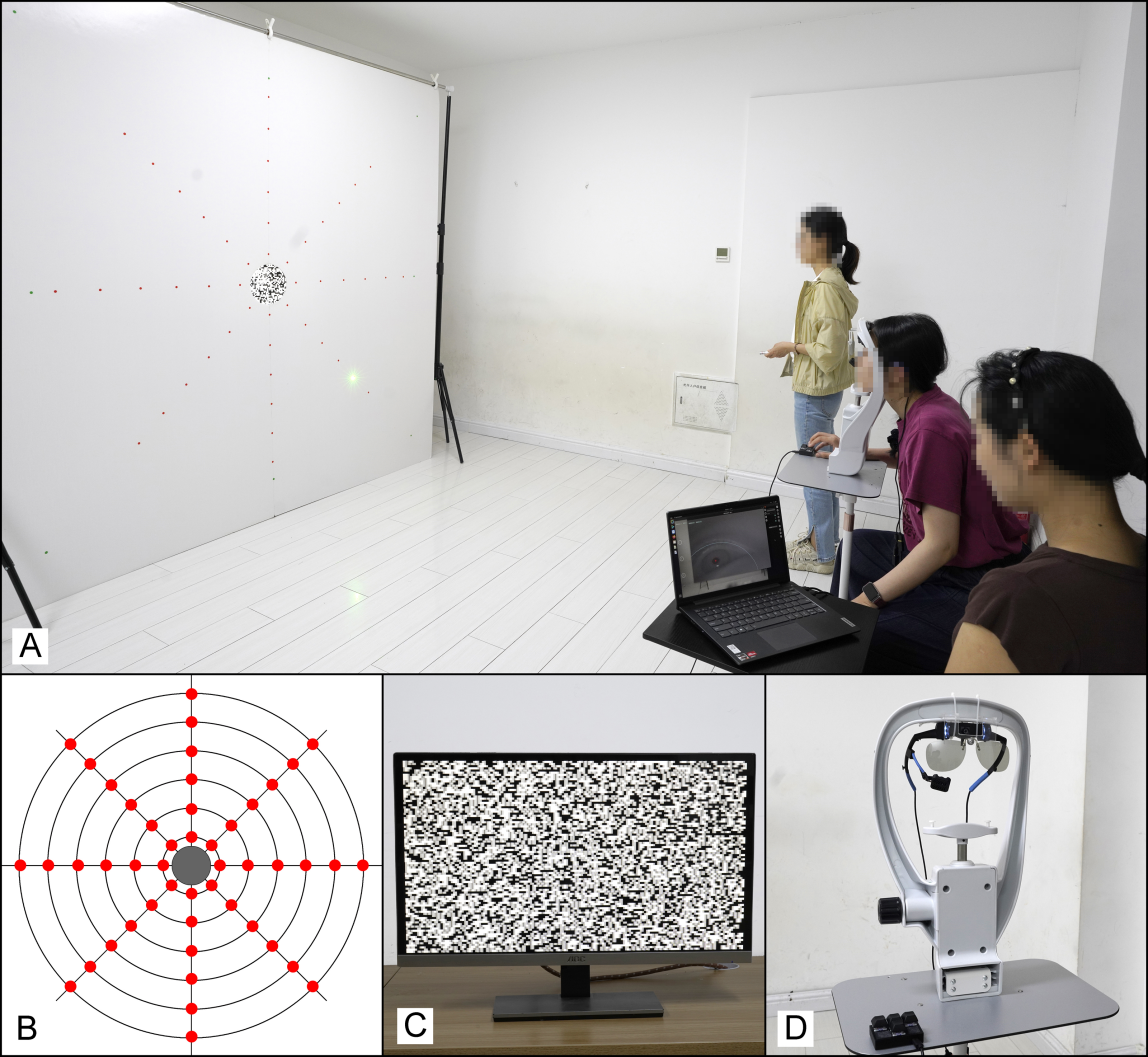
Diagram of the visual acuity and stereoacuity test system. (A) Diagram of the test setup. The test system comprised a polarized display positioned behind a 2 m × 2 m whiteboard. Participants were seated 3.4 m from the center of the whiteboard. An examiner (standing) used a laser pointer to indicate fixation points. Once the participant confirmed the position of the fixation point, the laser was turned off and was not turned on again during the test. The participant’s head was stabilized with a forehead and chin rest. Participants were instructed to maintain fixation on a designated test position while using their peripheral vision to observe a stimulus presented through a central aperture in a whiteboard. An examiner (seated diagonally behind) monitored and analyzed the eye-tracking data. (B) The whiteboard used for testing, with 48 fixation points marked. A 0.2 m central aperture allowed viewing of the monitor behind. (C) Polarized display examples. For stereoacuity testing, a random-dot stereogram was displayed. When wearing polarized lenses, participants could perceive a protruding stereoscopic symbol if their stereoacuity was better than the preset disparity. (D) A head-mounted eye tracker was attached to the upper portion of the forehead and chin rest. A polarizing filter was mounted in front of the eye tracker’s camera, which allowed the eye tracker to monitor eye movements directly without interference from the filter. Participants with refractive errors wore contact lenses during testing.

Stimuli were presented on a 23-inch polarized 3D display (AOC d2367PH, Admiral Overseas Co., Taiwan) with a resolution of 1,920  × 1,080 pixels and a screen refresh rate of 60 Hz. The mean luminance of the display was 47.7 cd/m^2^, measured using a luminance meter (SM208, Shenzhen Sanpo Instrument Co., Ltd., China) with the display showing a uniform neutral gray background. The test distance was set at 3.4 m, at which 0.06 m away from center correspond to 1° of retinal eccentricity. A whiteboard (2 m × 2 m) was constructed to measure VA and SVA in parafoveal and perifoveal retinal regions. The whiteboard included a circular aperture with a diameter of 0.20 m located at its center. The polarized display was positioned directly behind the aperture, allowing participants to view the presented stimuli. For VA testing, the display operated in normal mode. For SVA testing, the display switched to 3D mode, and participants wore polarized lenses (Fig. 1). Crosstalk of the polarized display system was measured at 1.15% following the methodology of Baker, Kaestner & Gouws (2016), ensuring adequate separation of left and right eye images during stereoacuity testing. Since stimuli were always presented at the center of the display while participants fixated on peripheral positions on the surrounding whiteboard, the viewing distance between the stimulus and the participant’s eyes did not change across test positions. For stereopsis testing, disparities were computationally defined by the pixel offset between the dichoptically presented left-eye and right-eye images.

Forty-eight test positions were set in total on the whiteboard. Using the center of the whiteboard as the reference origin, eight orientations were established: 0° (horizontal right), 45°, 90°, 135°, 180° (horizontal left), 225°, 270°, and 315°. These orientations formed four meridians: the horizontal meridian (0° and 180°), the vertical meridian (90° and 270°), and two oblique meridians (45° and 225°; 135° and 315°). Along each orientation, six test positions were placed from the center to the periphery. These positions corresponded to retinal eccentricities ranging from 2.5° to 15°, with increments of 2.5° (Fig. 1).

The Eyeso ED100 eye tracker (Braincraft Technology Co., Beijing, China) was used to monitor fixation stability during the VA tests and to prevent participants from shifting their gaze toward the center of the display. The eye tracker sampled at 100 Hz with a spatial accuracy of 0.5° and precision of 0.1°. Before testing began, a monocular 9-point calibration procedure was performed for the right eye of each participant to ensure accurate eye tracking. During testing, an experimenter continuously monitored the live gaze trace and equipment status in real time, interrupting and repeating any trial if obvious large saccades or loss of fixation were observed (Fig. 1). Subsequently, a more rigorous evaluation was performed using Eyeso Glasses software (V3.7). Fixation was considered stable when this position did not deviate more than 1° toward the center from the designated test position. If the deviation exceeded this threshold during any fixation point, the corresponding test positions were re-measured.

### Stimuli

**Figure 2:**
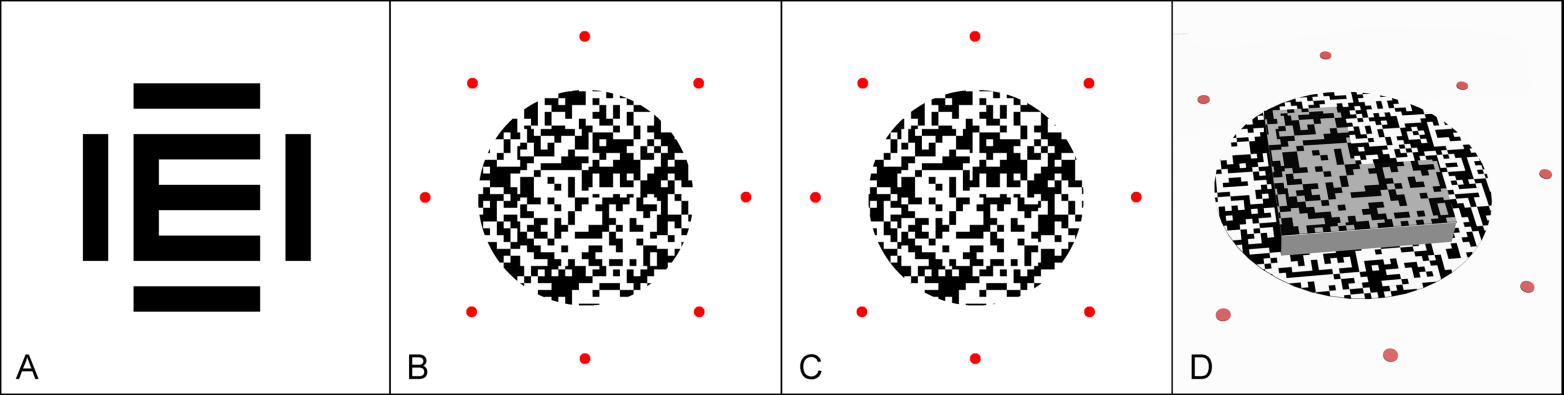
Stimuli used for visual acuity and stereoacuity testing. (A) E optotype with flankers, used for VA testing. (B) Image perceived by the right eye during SVA testing. (C) Image perceived by the left eye during SVA testing. (D) Simulated stereoscopic percept following proper fusion of (B) and (C).

All stimuli used in this study were generated using a program written in C++. To account for the crowding effect when identifying visual symbols, the tumbling E optotype with flankers, oriented in one of four possible directions was used for VA measurement (Fig. 2A). The size of optotype was calculated based on the test distance in 3.4 m, with pixel values of 4, 5, 6, 7, 9, 12, 15, 19, 24, 30, 37, 47, 59, and 75 corresponding to values ranging from 0 to 1.3 logarithm of the minimum angle of resolution (logMAR) in 0.1 logMAR increments.

The stereoscopic symbols were designed using a random dot pattern. The random dots were square-shaped to match the pixel characteristics of the display. Due to the reduced VA in the parafoveal and perifoveal regions, larger random dot sizes were employed to ensure that participants could correctly discern the stimuli. Random dot size varied with eccentricity and was calculated according to a model of acuity where the foveal threshold doubles at approximately 2.0° eccentricity (Anstis, 1974; Levi, Klein & Aitsebaomo, 1985; Strasburger, Rentschler & Jüttner, 2011). Dot size was set to 0.1 logMAR larger than the predicted values at each eccentricity to ensure that dots were clearly resolvable. This corresponded to dot sizes of approximately 12, 15, 24, and 30 pixels at 2.5°, 5°, 7.5°, and 10° eccentricity, respectively. Dot size was fixed across participants and limited to a maximum of 30 pixels for eccentricities of 10° and beyond to avoid introducing monocular form cues. The overall size of each stereoscopic symbol was determined by multiplying the size of a single random dot by a factor of 20. The stereoscopic symbols were designed with disparities of 1.7, 1.9, 2.1, 2.3, 2.5, 2.7, 2.9, 3.1, and 3.3 log arcsec, corresponding to 50″, 80″, 120″, 200″, 320″, 500″, 800″, 1,200″, and 2,000″, respectively.

The stereoscopic symbol was designed as a square with one quadrant removed (Figs. 2B, 2C, and 2D). This symbol was oriented in one of four possible directions: upper-right, upper-left, lower-right, or lower-left. During the stereopsis evaluation, participants were tasked to report the position of the missing quarter of the symbol. The traditional E symbol was not used due to limitations in pixel size. The stereoscopic symbol employed in this study was 480  × 480 pixels, and the maximum size of the random dots was 30  × 30 pixels. While participants could perceive the protruding stereoscopic symbol, it was difficult for them to identify the orientation of the E symbol. For a clear identification of the E symbol’s direction, a minimum size of 600  ×  600 pixels were required. In contrast, using a square with a missing quarter was effective at 240  ×  240 pixels, which was sufficient for participants to distinguish the missing corner. Therefore, a square with a missing corner was employed in this study.

### Measurement of binocular visual acuity

**Figure 3:**
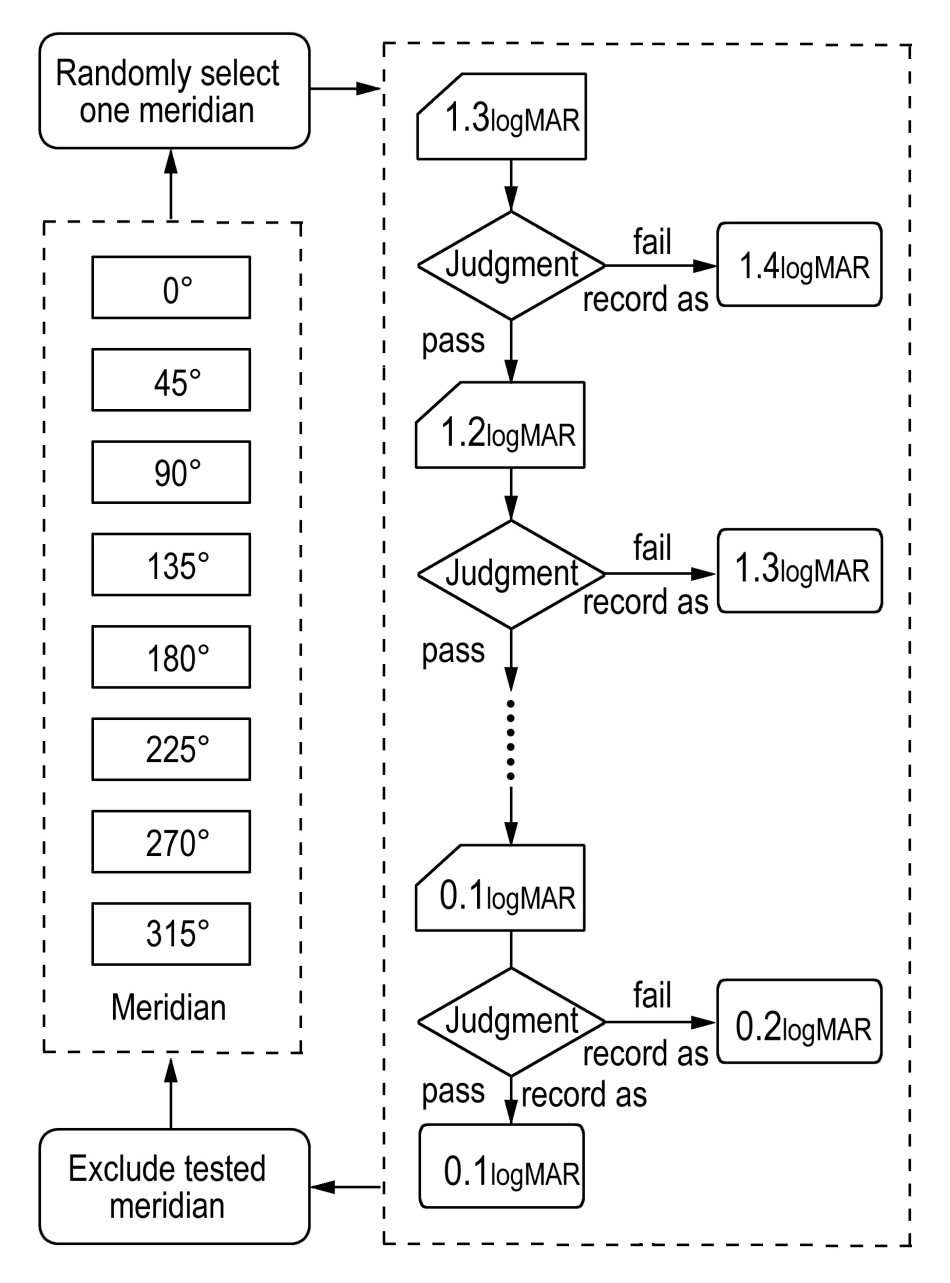
Flow chart of the visual acuity test procedure. One meridian was randomly selected from the eight meridians, and testing began at 1.3 logMAR. If the participant passed (≥3/5 correct) the test, the threshold decreased progressively until a failure occurred, at which point the last passed optotype size was recorded as the participant’s VA for this test position. If the participant failed at 1.3 logMAR, nil was recorded. After completing one meridian, it was excluded and the next meridian was randomly selected until all eight meridians were tested. This procedure was repeated at all six eccentricities, following the test sequence of 15°, 12.5°, 10°, 7.5°, 5°, and 2.5°. The numbers within the pentagon represent VA recorded in logMAR format.

The four-alternative forced choice task was employed to measure binocular VA. In this task, the tumbling E optotype with flankers, oriented in one of four possible directions (upper, lower, right, or left), was presented to the participant. The test began by measuring the test positions at 15° eccentricity regions. The participant was instructed to maintain fixation on one of eight randomly selected orientations. An optotype with a size of 1.3 logMAR was then displayed at the center of the display, and the participant was tasked to identify the direction of the optotype. If the participant did not respond within 10s, an incorrect result was recorded and the next stimulus was presented. The participant was required to correctly identify the optotype direction in at least three out of five presentations at the same symbol size to pass. After successful identification, the optotype size was reduced to 1.2 logMAR, and the test was repeated until the participant failed to correctly identify the optotype direction. The smallest optotype size that the participant successfully identified was recorded as their VA for this test position. The procedure was repeated for all 8 meridians at 15°, then for 12.5°, 10°, until 2.5°, resulting in 48 thresholds in total (Fig. 3).

Participants were instructed to rest for 1 min after every four test positions to minimize fatigue and maintain focus. The examiner monitored the participant’s eye movements in real time. If any saccades were detected during the test, the test was paused, and the participant was given a 30s rest period before the test continued. Following the measurement VA of all 48 test positions, the fixation stability was analyzed. Any tests that did not meet the predetermined stability criterion were recorded and re-measured. This process was repeated until stable fixation was achieved for all 48 positions.

### Measurement of stereopsis

The stereopsis test followed a similar procedure to the VA measurements with the four-alternative forced choice task and eccentricity order as the VA task, starting at 15° eccentricity regions and progressing centrally. Participants were instructed to maintain fixation on one of eight randomly selected orientations. A stereoscopic symbol with a disparity of 3.3 log arcsec was then presented at the center of the display, and participants were asked to report the position of the missing quarter of the square. Testing proceeded with stereoscopic symbols of incrementally smaller disparities following each correct identification. The test terminated when the participant either provided an incorrect response or failed to respond within the 10s. If the participant was unable to identify the stereoscopic symbol with a disparity of 3.3 log arcsec or did not respond, a SVA threshold of unmeasurable was recorded. The smallest disparity at which the participant correctly identified was recorded as their SVA. The procedure was repeated for the eight positions at 15° eccentricity, then for eight new test positions at 12.5°, and continued until all 48 test positions had been measured. The rest intervals for participants and the procedure for temporarily discontinuing the test, as described in the VA measurement procedure, were also followed during the stereopsis measurements.

### Statistical analysis

All analyses were conducted using R software (version 4.4.2) with the lme4 (version 1.1.37), lmerTest (version 3.1.3), geepack (version 1.3.12), and brms (version 2.22.0) packages. Unmeasurable VA and SVA values were recorded as nil, and the corresponding observations for that participant were excluded from the analysis. Generalized estimating equations (GEE) with an exchangeable correlation structure were used to analyze the influence of meridian and eccentricity on VA and SVA. Linear mixed-effects models (LMM) with subject-specific random intercepts were employed to predict VA and SVA based on eccentricity and meridian. The models included eccentricity, meridian direction, and their interaction as fixed effects, with random intercepts to account for between-subject variability. Model fitting was performed using restricted maximum likelihood (REML). A Bayesian mixed-effects approach was applied to address the ceiling effects observed in SVA. The 95% prediction intervals (95% PI) for the LMM were calculated in both fixed and random effects parameters as well as residual variance. Predictive performance for VA and SVA was evaluated using ten-fold cross-validation. A *P*-value less than 0.05 was considered statistically significant.

## Results

### Distribution of visual acuity in the parafoveal and perifoveal regions

**Figure 4:**
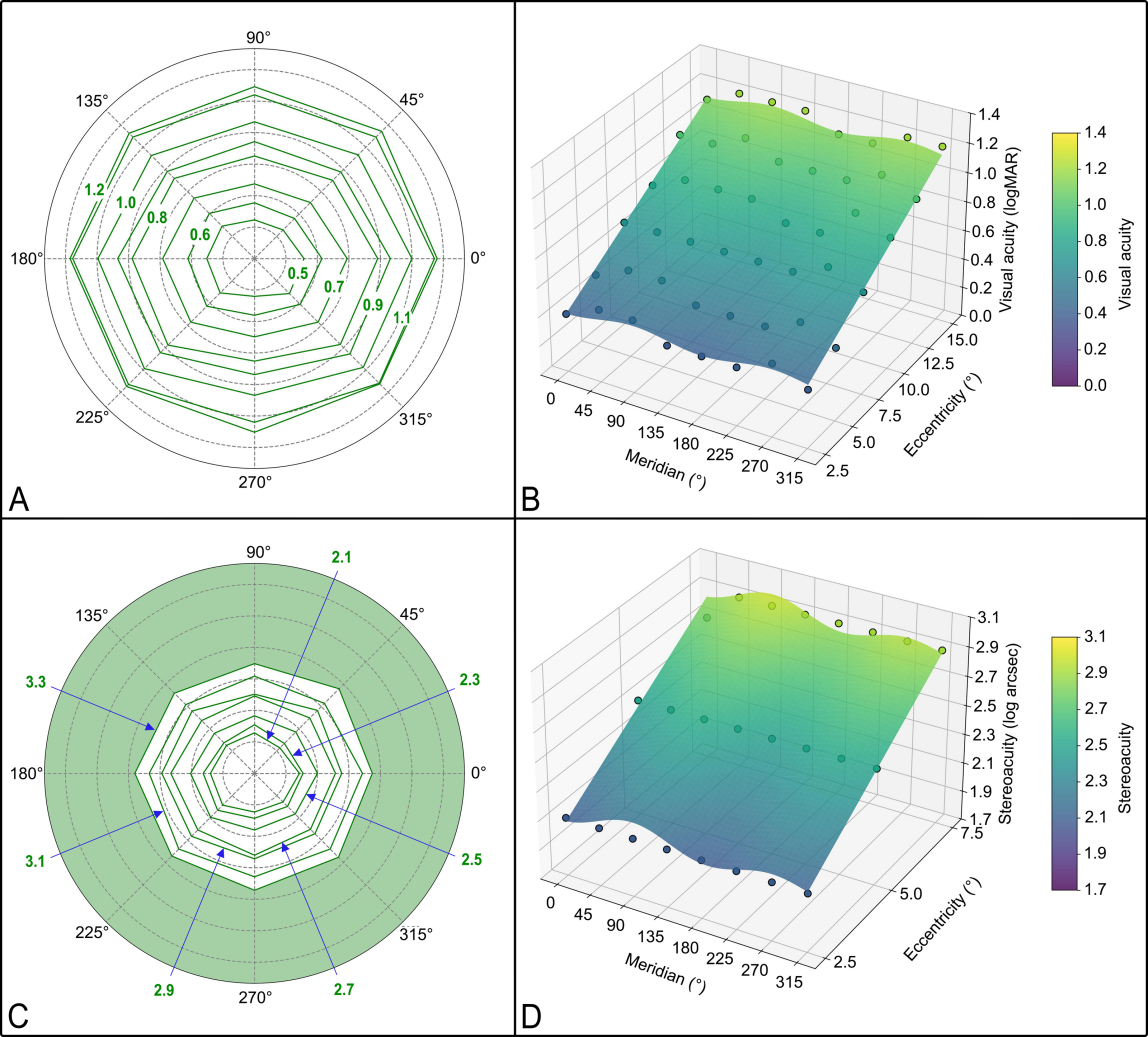
Isopters and predicted 3D plots of VA and SVA. (A) Isopters of VA, presented sequentially from 0. 5 to 1.2 logMAR in 0.1 logMAR increments. Concentric gray circles represented equal retinal eccentricities, with radii increasing by 2.5° from the fovea outward. (B) Predicted 3D plot of VA as a function of eccentricity and meridian. Color mapping indicated VA levels, with cooler colors indicating better VA and warmer colors indicating worse VA. Both predicted and observed VA demonstrate deterioration with increasing eccentricity. (C) Isopters of SVA. Each concentric gray circle represented a 2.5° increment in eccentricity from the fovea. At eccentricities of 10.0°, 12.5°, and 15.0°, median stereoacuity across all meridians was unmeasurable, indicating difficulty in recognizing stereoscopic symbols at these eccentricities. (D) Predicted 3D plot of SVA. Color mapping differentiated levels of stereoacuity, with cooler colors corresponding to better stereoacuity. Both predicted and observed stereoacuity deteriorated with increasing eccentricity.

Of the 1,680 tests performed by 35 participants, 32 tests (1.9%) resulted in failure to identify the optotype direction at the largest size tested. These failures occurred exclusively at eccentricities of 12.5° and 15.0°. The distribution of VA across eccentricities and meridians is summarized in Table 1. VA declined with increasing eccentricity, with the median of VA decreasing from 0.40 logMAR at 2.5° to 1.20 logMAR at 15.0°. Isopters, curves representing zones of equal VA across meridians (Low, 1951), are shown in Fig. 4A.

To account for the correlated nature of repeated measurements within subjects, we conducted a GEE analysis with an exchangeable correlation structure. The results revealed a significant main effect of eccentricity (*χ*^2^ = 1,168.317, *P* < 0.001) and meridian (*χ*^2^ = 85.684, *P* < 0.001) on VA. Specifically, VA differed significantly at meridians 45° (*χ*^2^ = 11.768, *P* = 0.001), 90° (*χ*^2^ = 18.796, *P* < 0.001), and 270° (*χ*^2^ = 14.449, *P* < 0.001), compared to 0°. The VA isopters illustrated that VA along the horizontal meridian was generally better than along the vertical meridian, which was similar to the GEE findings. The interaction between eccentricity and meridian was not statistically significant (*χ*^2^ = 9.669, *P* = 0.208), indicating that the rate of VA decline with eccentricity was similar across meridians.

### Predicting visual acuity based on eccentricity and meridian

The LMM was fitted using REML to predict VA. The full model included eccentricity and meridian as fixed effects, with subject-specific random intercepts to account for repeated measures. The meridian was modeled using the trigonometric terms sin*θ*, cos*θ*, and cos2*θ* to capture the circular periodicity of the visual field and its directional anisotropies. The results revealed a significant main effect of eccentricity (*β* = 0.057, *t* = 24.461, *P* < 0.001), indicating that VA decreased by approximately 0.057 logMAR per degree of eccentricity. The meridian of the first-order sine term (*β* = 0.012, *t* = 1.781, *P* = 0.075) and the first-order cosine term (*β* = 0.003, *t* = 0.518, *P* = 0.605) had no significant effect on VA. The meridian of the second-order cosine term showed a significant influence on VA (*β* =−0.029, *t* = −4.475, *P* < 0.001), which indicated that VA tends to be better along the horizontal meridians relative to the vertical meridians.

**Table 1:** Median (IQR) of VA (logMAR) across eccentricities and meridians.

Meridian	Eccentricity					
	2.5°	5.0°	7.5°	10.0°	12.5°	15.0°
0°	0.4 (0.2)	0.5 (0.1)	0.7 (0.1)	0.8 (0.1)	1.0 (0.1)	1.1 (0.1)
45°	0.5 (0.1)	0.6 (0.2)	0.7 (0.1)	0.9 (0.1)	1.0 (0.1)	1.2 (0.2)
90°	0.5 (0.1)	0.6 (0.2)	0.7 (0.2)	0.9 (0.2)	1.1 (0.2)	1.2 (0.1)
135°	0.4 (0.1)	0.5 (0.1)	0.7 (0.2)	0.9 (0.2)	1.0 (0.1)	1.2 (0.2)
180°	0.4 (0.2)	0.5 (0.1)	0.7 (0.2)	0.8 (0.2)	1.0 (0.1)	1.1 (0.1)
225°	0.4 (0.2)	0.5 (0.1)	0.7 (0.1)	0.8 (0.1)	1.0 (0.2)	1.1 (0.2)
270°	0.5 (0.1)	0.6 (0.1)	0.8 (0.1)	1.0 (0.2)	1.1 (0.1)	1.2 (0.1)
315°	0.4 (0.2)	0.5 (0.1)	0.7 (0.2)	0.9 (0.1)	1.0 (0.2)	1.2 (0.2)

The population-level prediction equations for VA were as follows:

(1) \begin{eqnarray*}\widehat{V{A}_{logMAR}}=0.290+0.057E-0.029\mathit{cos}~2\theta .\end{eqnarray*}

Here, ‘E’ represents eccentricity and ‘*θ*’ represents meridians in radians. The model explained a substantial proportion of variance, with marginal R^2^ values of 0.854. This indicated that the fixed effects accounted for 85.4% of the variance in VA. The between-subject variance (0.004) was smaller than the residual variance (0.007), suggesting consistent patterns of meridional anisotropy across participants. Individual deviations from population-level predictions were approximately 0.063 logMAR.

Ten-fold cross-validation at the subject level was used to assess the generalizability of the VA prediction model. The results showed a RMSE of 0.104 logMAR with a minimal systematic bias of −0.002 logMAR, supporting the utility of this formula for estimating peripheral VA in new subjects. The average cross-validation R^2^ was 0.836, indicating minimal overfitting. The predicted 3D plot of VA measurements generated using this model was shown in Fig. 4B.

### Distribution of stereoacuity in the parafoveal and perifoveal regions

In 792 of the 1,680 tests (47.14%) performed by all subjects, stereoacuity could not be measured at the largest disparity tested. This was rare at small eccentricities, 1.8% at 5.0° and 8.6% at 7.5°, but increased sharply at 10.0° (82.1%) and reached 98.2% at 15°. Given that stereoacuity could not be measured for most participants at eccentricities of 10.0°, 12.5° and 15.0°, only SVA measured within a 7.5 eccentricity range was used for quantitative analysis. The distribution of SVA across eccentricities and meridians is summarized in Table 2. SVA declined with increasing eccentricity.

**Table 2:** Median (IQR) of SVA (log arcsec) across eccentricities and meridians.

Meridian	Eccentricity		
	2.5°	5.0°	7.5°
0°	2.1 (0.4)	2.5 (0.4)	2.7 (0.4)
45°	2.1 (0.2)	2.5 (0.4)	2.9 (0.4)
90°	2.1 (0.4)	2.5 (0.6)	2.9 (0.6)
135°	2.1 (0.6)	2.5 (0.8)	2.9 (0.6)
180°	2.1 (0.4)	2.5 (0.4)	2.9 (0.6)
225°	2.1 (0.4)	2.5 (0.4)	2.9 (0.6)
270°	2.1 (0.6)	2.5 (0.6)	2.9 (0.8)
315°	2.1 (0.4)	2.5 (0.4)	2.9 (0.4)

The GEE analysis revealed a significant main effect of eccentricity (*χ*^2^ = 636.167, *P* < 0.001) and meridian (*χ*^2^ = 34.768, *P* < 0.001) on SVA. All meridians except 180° (*χ*^2^ = 0.140, *P* = 0.708) and 225° (*χ*^2^ = 0.170, *P* = 0.680) showed significant differences compared to 0°. The interaction between eccentricity and meridian was not statistically significant (*χ*^2^ = 1.950, *P* = 0.963). The isopters of SVA was shown in Fig. 4C.

### Predicting stereoacuity based on eccentricity and meridian

A Bayesian Tobit linear mixed-effects model with right-censoring was used to predict SVA. The results revealed a significant main effect of eccentricity on SVA (*β* = 0.154, *z* = 40.982, *P* < 0.001). For meridional effects, the first-order sine term was significant (*β* = 0.028, *z* =2.539, *P* = 0.011), indicating upper-lower asymmetry with superior meridian showing higher SVA than inferior meridian. The first-order cosine term was not significant (*β* =  − 0.003, *z* =  − 0.264, *P* = 0.792), indicating no significant difference in SVA between the left meridian and the right meridian. However, the second-order cosine term was significant (*β* =  − 0.053, *z* =  − 4.859, *P* < 0.001).

The population-level prediction equations for SVA for a given eccentricity and meridian were as follows:

(2) \begin{eqnarray*}\widehat{SV{A}_{\mathit{log}arcsec}}=1.745+0.154E+0.028\mathit{sin}~\theta -0.053\mathit{cos}~2\theta .\end{eqnarray*}

Here, ‘E’ represents eccentricity and ‘*θ*’ represents meridians in radians. The model explained substantial variance, with marginal R^2^ values of 0.596. The between-subject variance (0.025) was smaller than the residual variance (0.050), suggesting that stereopsis in the parafoveal and perifoveal regions may be less affected by individual differences.

Ten-fold cross-validation results showed a RMSE of 0.269 log arcsec with a systematic bias of −0.003 log arcsec. The average cross-validation R^2^ was 0.523, but still demonstrated reasonable predictive performance given the conservative nature of subject-level validation. The predicted 3D plot of SVA measurements generated using this model was shown in Fig. 4D.

### Correlation between visual acuity and stereopsis

Table 3 shows the correlation between SVA and VA at each test position. Although both SVA and VA decreased with increasing eccentricity, Spearman rank correlation analysis revealed no significant correlations were observed at all test positions within 7.5° eccentricities (*P* > 0.05).

## Discussion

This study quantifies binocular VA and SVA in young subjects. Both VA and SVA deteriorated with increasing retinal eccentricity. However, the decline was about threefold steeper for SVA (0.154 log arcsec per degree) than for VA (0.057 logMAR per degree). Consistent horizontal-over-vertical advantages were observed, with the horizontal meridian supporting 0.058 logMAR better VA and 0.106 log arcsec better SVA than the vertical meridian. These findings align with the study of Fendick & Westheimer (1983), which observed similar deterioration patterns for both VA and stereopsis from the fovea to 10° eccentricity along the horizontal and vertical meridians.

### Visual acuity across eccentricities and meridians

Within the 2.5°–15° range, binocular VA deteriorated steadily at approximately 0.057 logMAR per degree. The horizontal meridian VA was 0.058 logMAR better than the vertical VA within 12.5° eccentricities, similar to the horizontal-over-vertical anisotropy previously reported using gratings and orientation discrimination tasks (Barbot, Xue & Carrasco, 2021; Carrasco, Williams & Yeshurun, 2002).

**Table 3:** Spearman rank correlation test between SVA and VA by meridian and eccentricity.

Meridian	Eccentricity					
	2.5°		5.0°		7.5°	
	*r*_*s*_	*P*	*r*_*s*_	*P*	*r*_*s*_	*P*
0°	−0.087	0.620	−0.131	0.453	−0.237	0.171
45°	0.079	0.650	0.107	0.542	0.196	0.258
90°	0.082	0.638	−0.066	0.707	0.037	0.835
135°	0.091	0.604	0.102	0.561	−0.034	0.846
180°	−0.090	0.606	0.055	0.753	−0.131	0.452
225°	0.077	0.662	0.210	0.227	0.149	0.393
270°	0.074	0.673	0.121	0.488	−0.327	0.055
315°	0.162	0.353	−0.174	0.319	−0.034	0.848

The decline in VA across the paracentral field reflects changes in both retinal cone density and cortical visual processing. At the population level, the average cone density decrease of about 50% from 2.5° to 15° appears insufficient to account for the observed VA decline, which corresponded to a median eightfold increase in symbol size over this range. It should be noted that cone density varies among individuals, and such inter-individual variation may contribute to the between-subject variance observed in our study. Nevertheless, the magnitude of VA decline exceeds what photoreceptor decline alone would predict, suggesting that factors beyond photoreceptor distribution may dominate paracentral VA loss. Previous studies have shown that midget retinal ganglion cell density and cortical magnification decrease more steeply with eccentricity than cone density, and receptive fields in primary visual cortex (V1) approximately double in width every 6°, resulting in greater neural pooling and reduced spatial resolution (Fendick & Westheimer, 1983; He, Wang & Fang, 2019; Heitmann et al., 2023). Moreover, equivalent noise modeling indicated that beyond 10° eccentricity, performance for fine symbols is limited more by internal neural noise than by sampling density (Masri, Grünert & Martin, 2020). The persistent horizontal advantage likely reflects higher ganglion cell density and a larger cortical surface area devoted to processing the horizontal meridian, potentially amplified by attentional biases favoring the horizontal visual field (Barbot, Xue & Carrasco, 2021; Himmelberg, Winawer & Carrasco, 2023).

### Stereoacuity across eccentricities and meridians

Stereo thresholds increased steeply with retinal eccentricity, increasing from a median of 2.1 log arcsec at 2.5° to 2.9 log arcsec at 7.5°. From 10° eccentricity, more than half of the SVA measurements could not be obtained, demonstrating the rapid loss of depth precision in the peripheral retina. Across the measurable range, the horizontal meridian supported stereopsis that was 0.106 log arcsec finer than the vertical meridian. Psychophysical equivalent noise analysis revealed that a fivefold increase in early internal disparity noise dominates peripheral performance (Wardle et al., 2012), while single-unit recordings in macaque V1 demonstrated systematic broadening of disparity-tuned receptive fields over a range of 0.5°–5° eccentricity (Prince, Cumming & Parker, 2002). Furthermore, psychophysical studies showed that stereoacuity deterioration with eccentricity depends on stimulus spatial frequency content (Siderov & Harwerth, 1995). Stereo thresholds for low-spatial-frequency stimuli remain relatively invariant across the visual field, whereas thresholds for high-spatial-frequency stimuli deteriorate rapidly. In our study, the fundamental spatial frequencies of dot sizes remained relatively high. The observed decline in stereoacuity suggested that despite scaling, the visual system’s sensitivity to the broadband content of random-dot stereograms may still be limited by these high-frequency constraints and the associated increase in internal noise observed for broadband stimuli (Wardle et al., 2012), rather than exhibiting the invariance characteristic of low-frequency mechanisms. Human fMRI population receptive field mapping has corroborated this cortical pooling mechanism and revealed both larger cortical surface area and finer sampling for the horizontal *versus* vertical visual field, explaining the observed horizontal-over-vertical advantage (Himmelberg, Winawer & Carrasco, 2023). These retinal and cortical constraints account for the steep and anisotropic decline in stereopsis observed in our study.

### Relationship between visual acuity and stereoacuity

Previous studies examining the relationship between VA and SVA have primarily focused on patients with macular diseases. In patients with a unilateral macular hole or epiretinal membrane, stereo thresholds increase proportionally to the acuity deficit (Asaria et al., 2008; Hikichi et al., 2001). However, our study found no correlation between VA and SVA in the healthy paracentral retina, suggesting these functions depend on distinct neural mechanisms. Paracentral VA is primarily limited by reduced photoreceptor and midget retinal ganglion cell density, whereas stereopsis deteriorates more rapidly due to a fivefold increase in internal disparity noise and expansion of disparity-tuned receptive fields in V1/V2 (Prince, Cumming & Parker, 2002; Wardle et al., 2012). To minimize stereopsis dependence on spatial resolution, we employed large-dot random-dot stereograms with dot sizes 4–8 times larger than the local Nyquist limit. This design ensured that participants with reduced paracentral VA could still resolve the stereo stimuli, preventing VA from becoming a rate-limiting factor for stereopsis performance. Our results showed that random-dot stereograms can still elicit measurable stereopsis in the paracentral field despite moderate VA loss. However, stereopsis declined approximately three times more rapidly than VA with increasing eccentricity.

### Prediction equations and potential application

The censored mixed-effects models described above can be expressed as equations that generate both a predicted threshold and its 95% PI. For instance, at 4.5° eccentricity on the 160° meridian, the predicted SVA was 2.40 log arcsec (95% PI [1.87–2.95] log arcsec) and the predicted VA was 0.53 logMAR (95% PI [0.33–0.73] logMAR). Therefore, the VA and SVA from healthy young subjects have a 95% probability of falling within these prediction intervals. Given the inherent sensitivity of SVA measurements to stimulus and apparatus characteristics, the reference values and prediction equations presented here should be applied with caution when using different stereotests.

Verghese, Ghahghaei & Lively (2022) demonstrated that patients with macular degeneration exhibit stereopsis loss within lesion areas while retaining residual stereopsis in unaffected regions. Our study aimed to complement these findings by providing reference prediction equations that transform individual patient measurements into interpretable functional deviation maps. In clinical practice, structural OCT imaging of lesions can be spatially registered to our reference polar grid, enabling direct comparison of a patient’s measured VA or SVA thresholds at specific lesion coordinates against reference predictions. This comparison could reveal deviations attributable to pathology. By aggregating these deviations across patient cohorts, clinicians can objectively quantify disease-specific alterations in the spatial patterns of VA and SVA loss relative to age-matched norms. Furthermore, these equations can be incorporated into clinical software to enable real-time identification of abnormal retinal positions and inform personalized rehabilitation strategies. For example, the system could guide patients in repositioning their preferred retinal locus toward areas predicted to offer superior visual performance. While our current reference values derive from healthy adults aged 22–33 years, this predictive framework is designed to accommodate future updates incorporating measurements from older populations or specific ocular conditions.

### Limitations and future directions

This study has several limitations that should be considered. First, the majority of participants were restricted to healthy adults aged 22–33 years, which is younger than the high incidence age of macular damage observed in clinical practice, so the prediction equations should be re-estimated for older populations in future. Second, the range of VA and stereopsis measurements in this study was between 2.5° and 15.0° from the foveal center, which did not include measurements within the 0° to 2.5° interval. Future studies should consider incorporating measurements closer to the foveal center to expand the dynamic range. Third, all 35 participants had normal binocular vision, which may limit the applicability of the prediction equations in individuals with binocular vision anomalies. Future studies could investigate how conditions such as anisometropia influence the relationship between eccentricity, meridian, and visual function.

## Conclusion

Both VA and SVA deteriorate with increasing eccentricity, with stereopsis declining about three times faster and demonstrating a more pronounced horizontal-over-vertical advantage. After accounting for eccentricity and meridian, VA and SVA were essentially uncorrelated, suggesting distinct neural mechanisms underlying these functions. Prediction equations with 95% PI were developed for VA and SVA, establishing reference benchmarks for healthy adults in the paracentral field. These equations facilitate clinical interpretation of patient measurements and provide a new perspective for future studies of disease-related VA and SVA changes.

##  Supplemental Information

10.7717/peerj.21251/supp-1 Supplemental Information 1 Test results of stereocuity (log arcsec) under different test positions

10.7717/peerj.21251/supp-2 Supplemental Information 2 Test results of vsual acuity (logMAR) under different test positions

10.7717/peerj.21251/supp-3 Supplemental Information 3 STROBE Checklist for Cross-Sectional Studies - completed reporting guideline

## Additional Information and Declarations

### Data Availability

The following information was supplied regarding data availability: The data is available in the Supplemental Files.

## References

[ref-1] Anstis SM (1974). Chart demonstrating variations in acuity with retinal position. Vision Research.

[ref-2] Asaria R, Garnham L, Gregor ZJ, Sloper JJ (2008). A prospective study of binocular visual function before and after successful surgery to remove a unilateral epiretinal membrane. Ophthalmology.

[ref-3] Baker DH, Kaestner M, Gouws AD (2016). Measurement of crosstalk in stereoscopic display systems used for vision research. Journal of Vision.

[ref-4] Barbot A, Xue S, Carrasco M (2021). Asymmetries in visual acuity around the visual field. Journal of Vision.

[ref-5] Blakemore C (1970). The range and scope of binocular depth discrimination in man. The Journal of Physiology.

[ref-6] Bosten J, Goodbourn P, Lawrance-Owen A, Bargary G, Hogg R, Mollon J (2015). A population study of binocular function. Vision Research.

[ref-7] Carrasco M, Williams PE, Yeshurun Y (2002). Covert attention increases spatial resolution with or without masks: support for signal enhancement. Journal of Vision.

[ref-8] Davson H (1990). Visual acuity. Physiology of the eye.

[ref-9] De Lestrange-Anginieur E, Kee CS (2020). Investigation of the impact of blur under mobile attentional orientation using a vision simulator. PLOS ONE.

[ref-10] Dormegny L, Foch M, Messerlin A, Bourcier T, Sauer A, Gaucher D (2023). Binocular visual function improvement after pars plana vitrectomy for epiretinal membrane. Acta Ophthalmologica.

[ref-11] Fendick M, Westheimer G (1983). Effects of practice and the separation of test targets on foveal and peripheral stereoacuity. Vision Research.

[ref-12] Ghahghaei S, McKee S, Verghese P (2019). The upper disparity limit increases gradually with eccentricity. Journal of Vision.

[ref-13] He D, Wang Y, Fang F (2019). The critical role of V2 population receptive fields in visual orientation crowding. Current Biology.

[ref-14] Heitmann C, Zhan M, Linke M, Hölig C, Kekunnaya R, Van Hoof R, Goebel R, Röder B (2023). Early visual experience refines the retinotopic organization within and across visual cortical regions. Current Biology.

[ref-15] Hikichi T, Onodera A, Ishiko S, Fujio N, Mori F, Yoshida A (2001). Stereo acuity in patients with unilateral macular hole and after unilateral macular hole surgery. Graefe’s Archive for Clinical and Experimental Ophthalmology.

[ref-16] Himmelberg MM, Winawer J, Carrasco M (2023). Polar angle asymmetries in visual perception and neural architecture. Trends in Neurosciences.

[ref-17] Howard IP, Rogers BJ (2012). Perceiving in depth, volume 2: stereoscopic vision.

[ref-18] Lam AK, Chau AS, Lam W, Leung GY, Man BS (1996). Effect of naturally occurring visual acuity differences between two eyes in stereoacuity. Ophthalmic and Physiological Optics.

[ref-19] Levi DM, Klein SA, Aitsebaomo A (1985). Vernier acuity, crowding and cortical magnification. Vision Research.

[ref-20] Levy NS, Glick EB (1974). Stereoscopic perception and Snellen visual acuity. American Journal of Ophthalmology.

[ref-21] Liu L, Xu L, Yu B, Zhao L, Wu H (2024). The influence of simulated visual impairment on distance stereopsis. Journal of Vision.

[ref-22] Low FN (1951). Peripheral visual acuity. AMA Archives of Ophthalmology.

[ref-23] Masri RA, Grünert U, Martin PR (2020). Analysis of parvocellular and magnocellular visual pathways in human retina. Journal of Neuroscience.

[ref-24] Mochizuki H, Shoji N, Ando E, Otsuka M, Takahashi K, Handa T (2012). The magnitude of stereopsis in peripheral visual fields. Kitasato Medical Journal.

[ref-25] Okamoto F, Morikawa S, Moriya Y, Sugiura Y, Murakami T, Tomioka M, Hiraoka T, Oshika T (2020). Vision-related parameters that affect stereopsis in patients with macular hole. Scientific Reports.

[ref-26] Okamoto F, Tomioka M, Murakami T, Morikawa S, Sugiura Y, Hiraoka T, Oshika T (2021). Relationship between stereopsis and vision-related quality of life following intravitreal ranibizumab injections for central retinal vein occlusion. Scientific Reports.

[ref-27] Prince SJ, Cumming BG, Parker AJ (2002). Range and mechanism of encoding of horizontal disparity in macaque V1. Journal of Neurophysiology.

[ref-28] Rawlings SC, Shipley T (1969). Stereoscopic acuity and horizontal angular distance from fixation. Journal of the Optical Society of America.

[ref-29] Rees A, Kabanarou S, Culham L, Rubin G (2005). Can retinal eccentricity predict visual acuity and contrast sensitivity at the PRL in AMD patients?. International Congress Series.

[ref-30] Rovamo J, Virsu V (1979). An estimation and application of the human cortical magnification factor. Experimental Brain Research.

[ref-31] Shipley T, Popp M (1972). Stereoscopic acuity and retinal eccentricity. Ophthalmic Research.

[ref-32] Siderov J, Harwerth RS (1995). Stereopsis, spatial frequency and retinal eccentricity. Vision Research.

[ref-33] Sitko KR, Peragallo JH, Bidot S, Biousse V, Newman NJ, Bruce BB (2016). Pitfalls in the use of stereoacuity in the diagnosis of nonorganic visual loss. Ophthalmology.

[ref-34] Smith AT, Singh KD, Williams AL, Greenlee MW (2001). Estimating receptive field size from fMRI data in human striate and extrastriate visual cortex. Cerebral Cortex.

[ref-35] Strasburger H, Rentschler I, Jüttner M (2011). Peripheral vision and pattern recognition: a review. Journal of Vision.

[ref-36] Swanson WH, Fish GE (1995). Color matches in diseased eyes with good acuity: detection of deficits in cone optical density and in chromatic discrimination. Journal of the Optical Society of America. A, Optics, Image Science, and Vision.

[ref-37] Taipale J, Mikhailova A, Ojamo M, Nättinen J, Väätäinen S, Gissler M, Koskinen S, Rissanen H, Sainio P, Uusitalo H (2019). Low vision status and declining vision decrease health-related quality of life: results from a nationwide 11-year follow-up study. Quality of Life Research.

[ref-38] Verghese P (2023). The utility of peripheral stereopsis. Frontiers in Neuroscience.

[ref-39] Verghese P, Ghahghaei S, Lively Z (2022). Mapping residual stereopsis in macular degeneration. Journal of Vision.

[ref-40] Wardle SG, Bex PJ, Cass J, Alais D (2012). Stereoacuity in the periphery is limited by internal noise. Journal of Vision.

